# Postmenopausal women’s perceptions regarding menopause within the context of cognitive behavioral model: A qualitative evaluation

**DOI:** 10.1192/j.eurpsy.2023.2394

**Published:** 2023-07-19

**Authors:** F. Atkan, F. Oflaz

**Affiliations:** 1 Psychiatric Nursing, Koç University, Istanbul, Türkiye

## Abstract

**Introduction:**

Women experience some physiological, cognitive and emotional symptoms due to changes during menopause, which is a critical period in their lives. Every woman experience differences in symptoms and severity of them. Women’s individual perceptions and attitudes towards menopause affect their quality of life.

**Objectives:**

The aim of this study is to describe women’s perceptions and feelings about menopausal process and symptoms experienced and their behaviours to cope with these symptoms within framework of Cognitive Behavioral Model.

**Methods:**

Phenomenology design was used in this study.Three focus group interviews were held June-September 2022, using Zoom platform.Purposive sampling was used as the sampling method of study.A total of 13 women in a menopausal period of 1-15 years, 5-6 people in each group, were included in the study.Focus group interviews were held for 1-1.5 hours and once with each group. In the focus group interviews, a ‘Semi-Structured Interview Form’ consisting of 4 questions was used to evaluate perceptions, changes and behaviors of postmenopausal women regarding menopause process.Zoom recordings were taken during the interviews and data were written down.The data were evaluated by thematic analysis method within framework of Cognitive Behavioral Model.

**Results:**

In this study, 11 themes were defined as women’s perception of menopause at individual and social level.These themes are negative automatic beliefs about female identity such as menopause reduces woman and loses her femininity, woman is not understood and struggles alone, woman has been stigmatized, women need support, hidden and spoken in a low voice, long and difficult process, also beliefs that physiological changes occur in the body associated with the fact that it’is a natural process and that it’is necessary to use new coping strategies to effectively manage these changes, onset of diseases, changing relationships, and high self-observation process.Emotions such as irritability, tension, sadness, and hypersensitivity are negative automatic beliefs that decrease woman and loss of femininity, accompanied by emotions such as irritability, sadness, and behaviors such as crying crises, social withdrawal, that woman is not understood and is a process that she struggles alone, avoidance and social withdrawal behaviors such as reading books, listening music, walking alone.

**Conclusions:**

In this study, it was observed that women had difficulties with gender identity along with physiological and psychological changes during menopause, and there was an increase in self-observation.It was determined that they used some new behavioral and psychological strategies to cope with this new situation.It was evaluated that these changes in emotions, thoughts and behaviors could be well formulated within the cognitive behavioral model and this model would be useful in supporting women.

**Disclosure of Interest:**

None Declared

